# Implicit Learning in Transient Global Amnesia and the Role of Stress

**DOI:** 10.3389/fnbeh.2016.00222

**Published:** 2016-11-17

**Authors:** Frauke Nees, Martin Griebe, Anne Ebert, Michaela Ruttorf, Benjamin Gerber, Oliver T. Wolf, Lothar R. Schad, Achim Gass, Kristina Szabo

**Affiliations:** ^1^Department of Cognitive and Clinical Neuroscience, Medical Faculty Mannheim, Central Institute of Mental Health, Heidelberg UniversityMannheim, Germany; ^2^Department of Neurology, Universitätsmedizin Mannheim, Heidelberg UniversityMannheim, Germany; ^3^Computer Assisted Clinical Medicine, Medical Faculty Mannheim, Heidelberg UniversityMannheim, Germany; ^4^Department of Cognitive Psychology, Institute of Cognitive Neuroscience, Ruhr-University BochumBochum, Germany

**Keywords:** aversive conditioning, context, hippocampus, striatum, transient global amnesia

## Abstract

Transient global amnesia (TGA) is a disorder with reversible anterograde disturbance of explicit memory, frequently preceded by an emotionally or physically stressful event. By using magnetic resonance imaging (MRI) following an episode of TGA, small hippocampal lesions have been observed. Hence it has been postulated that the disorder is caused by the stress-related transient inhibition of memory formation in the hippocampus. In experimental studies, stress has been shown to affect both explicit and implicit learning—the latter defined as learning and memory processes that lack conscious awareness of the information acquired. To test the hypothesis that impairment of implicit learning in TGA is present and related to stress, we determined the effect of experimental exposure to stress on hippocampal activation patterns during an implicit learning paradigm in patients who suffered a recent TGA and healthy matched control subjects. We used a hippocampus-dependent aversive learning procedure (context conditioning with the phases habituation, acquisition, and extinction) during functional MRI following experimental stress exposure (socially evaluated cold pressor test). After a control procedure, controls showed successful learning during the acquisition phase, indicated by increased valence, arousal and contingency ratings to the paired (CON+) vs. the non-paired (CON−) conditioned stimulus, and successful extinction of the conditioned responses. Following stress, acquisition was still successful, however extinction was impaired with persistently increased contingency ratings. In contrast, TGA patients showed impairment of conditioned responses and insufficient extinction after the control procedure, indicated by a lack of significant differences between CON+ and CON− for valence and arousal ratings after the acquisition phase and by significantly increased contingency ratings after the extinction. After stress, aversive learning was not successful with non-significant ratings of all parameters. Concerning brain activation patterns after the control procedure, controls showed increased hippocampal response during acquisition after the control procedure. This was not seen after stress exposure. In TGA patients, we observed an increased response in the right ventral striatum in the acquisition phase following stress. These findings suggest that alterations in implicit learning processes, including impaired hippocampal and increased striatal responses, might play a role in TGA pathophysiology, partly related to acute stress.

## Introduction

Transient global amnesia (TGA) is an acute amnestic syndrome, which is characterized by a sudden disturbance of anterograde episodic long-term memory and a disruption of learning novel episodic information that usually resolves within 24 h (Hodges and Warlow, 1990; Jäger et al., 2009). Several groups have suggested that the pathophysiological mechanisms leading to TGA might be similar to those of cerebral ischemia, epilepsy or migraine (Frederiks, 1993), or might be a consequence of a disturbance of venous hemodynamics (Baracchini et al., 2012). However, there is no definitive evidence supporting any of these mechanisms.

Interestingly, the observation that physically or emotionally stressful events precede the amnestic episode has led to the implication that stress might trigger TGA (Quinette et al., 2006). More precisely, it has been hypothesized that TGA might be caused by a stress-related transient inhibition of memory formation in the hippocampus due to a selective vulnerability of CA-1 neurons to metabolic stress (Bartsch and Deuschl, 2010). In up to 80% of patients, diffusion-weighted MRI detects small lesions in the hippocampus 24–48 h after symptom onset (Sedlaczek et al., 2004), corroborating the anatomical link of the disorder to the hippocampal CA-1 subfield, without testifying its etiology. Moreover, changes in the endocrinal stress regulation system might also play a role. We found a stronger reduction of cortisol levels following pharmacological suppression of the hypothalamic-pituitary-adrenal (HPA) axis with dexamethasone and stronger response of cortisol in anticipation of an experimental stressor in individuals with a history of recent TGA, which might indicate a hypersensitivity of the HPA axis (Griebe et al., 2015). The clinical features of and MRI findings in TGA patients support the assumption of a stress-related etiology, due to the fact that glucocorticoids have a high concentration in the hippocampus, a central region in learning and memory processes (Lang et al., 2009), whose structural plasticity has been demonstrated to be impaired as a consequence of stress (McEwen, 2001).

Previous studies however, have not only demonstrated impairments in explicit (Merz et al., 2012) but also implicit learning and memory processes (Merz et al., 2013a) following stress exposure or with relation to the stress hormone cortisol. While explicit memory impairment is the main feature of TGA, the involvement of implicit learning and memory processes and whether they are related to differences in stress processing has not been studied.

One of the simplest and best-studied implicit learning mechanisms is Pavlovian conditioning. Here, a predictive association between a neutral stimulus (the conditioned stimulus or CS) and a biologically meaningful signal (the unconditioned stimulus or US) is learned. After one or several pairings of CS and US (during the acquisition phase), the CS alone elicits a fear/aversive response (the conditioned response, CR) (LeDoux, 2003). In the absence of further reinforcement, the conditioned responses gradually subside (extinction phase). Previous studies have shown that increased levels of the HPA axis stress hormone cortisol are associated with enhanced fear anticipation and impaired extinction of conditioned fear responses (Jackson et al., 2006; Merz et al., 2013a). These alterations of learning and memory by stress, in particular those mediated by glucocorticoids, probably reflect effects in limbic brain regions such as the amygdala (Roozendaal et al., 2009) and in particular the hippocampus (McEwen, 2001). While for Pavlovian cue conditioning, the hippocampus is not a target region, the acquisition during context conditioning critically depends on hippocampal function—demonstrated both in animals, as derived from lesion studies in rodents (Phillips and LeDoux, 1992), and in humans using functional magnetic resonance imaging (fMRI) and positron emission tomography studies (Lang et al., 2009).

This study was designed to test the hypothesis that impairment of implicit learning in TGA is present and is related to stress. Therefore, we investigated TGA patients and a matched control group, who completed a contextual aversive conditioning paradigm during fMRI, including both acquisition and extinction phases after an experimental stress induction and a control procedure. The contextual paradigm was used due to its dependence on the hippocampus (Lang et al., 2009), as also stated above, which allows to investigate implicit learning alterations along hippocampal functions. As stressor, we used the socially evaluated cold pressor test (SECPT) and used warm water for the control condition. The SECPT has previously been established to cause a substantial increase of cortisol values (Schwabe et al., 2008). This is related to the release of glucocorticoids that has been demonstrated to influence hippocampal functions (Schwabe and Wolf, 2012).

We expected individuals with a history of TGA to show similar aversive conditioning as the control group in the control condition, without significant differences in brain activations and subjective learning parameters of valence, arousal and contingency ratings. However, after the SECPT, TGA patients are hypothesized to feature stress-induced impairments in fear acquisition and extinction indicated by reduced brain activation, particularly in the hippocampus, as well as reduced differential conditioned responses on a subjective level.

## Materials and methods

### Participants

The sample is identical to our study on neuroendocrine findings in TGA (Griebe et al., 2015). From our prospectively collected TGA database with 208 cases since 2001 fulfilling the established criteria (Hodges and Warlow, 1990), we contacted those 41 who had experienced a TGA in the years 2010 and 2011. We included 20 right-handed TGA patients (*n* = 8 male) and 20 controls (*n* = 8 male) matched for age, sex and education (see Table 1). Exclusion criteria were: left-handedness, medication, or comorbidity interfering with the conduct of the trial (e.g., tremor). The study examinations were performed on 3 days: on day 1 (in the morning), the neuropsychological evaluation and self-report scales of depression, anxiety and stress; on day 2 and day 3 (1 week apart at the same time of day, between 1 and 4 pm), the SECPT or control procedure in a randomized order followed by the MRI examination (details see below). State and trait anxiety were measured using the German version of the State-Trait Anxiety Inventory (STAI; Laux et al., 1981), depression using the German version of the General Depression Scale (ADS; Hautzinger and Bailer, 1993), and chronic stress using the Trier Inventory for Chronic Stress (TICS; Schulz and Schlotz, 1999). For all questionnaires the individual mean scores were computed (see Table 1). The study was approved by the Ethics Committee of the Medical Faculty Mannheim, Heidelberg University and conformed to the Code of Ethics of the World Medical Association (Declaration of Helsinki, 6th revision, 2008). Written informed consent was obtained before the experiment.

**Table 1:** **Demographic information and findings on depression, anxiety, and chronic stress**.

	**TGA**	**Control group**	***p*-value**
Age, years; mean (SD)	66.50 (7.70)	66.55 (7.00)	n.s.
Formal education, years; median (range)	12.5 (8–19)	13 (8–17)	n.s.
Depression, ADS; mean (SEM)	12.30 (5.17)	8.50 (6.22)	0.021
Anxiety, STAI; mean (SEM)	39.30 (9.00)	31.00 (9.50)	0.007
Chronic stress, TICS; mean (SEM)	67.15 (3.67)	59.2 (3.16)	n.s.

*n.s., non-significant; SD, standard deviation; SEM, standard error of the mean; TGA, transient global amnesia; ADS, General Depression Scale; STAI, State-Trait Anxiety Inventory; TICS, Trier Inventory for Chronic Stress*.

### Stress exposure: socially evaluated cold pressor test (SECPT)

All participants were exposed to the SECPT (Schwabe et al., 2008). They were advised to immerse their right hand including the wrist in ice water (0–4°C) for 3 min or until they could no longer tolerate it. During the procedure, they were supervised by an unfamiliar person and informed that they were being videotaped for analysis of facial expression. For the control procedure, we used warm water (35–37°C) and participants were not filmed. Each participant underwent both the SECPT and the control procedure, performed in a randomized order across individuals. Immediately after the SECPT and the control procedure, participants were asked to rate how stressful, painful, and unpleasant the experiment had been (0 “not at all” to 100 “very”). Blood pressure (auscultatory method with stethoscope and aneroid sphygmomanometer), heart rate (manual palpation of radial artery), and salivary cortisol values were collected before, after 3 min and after 15 min (Griebe et al., 2015).

### Paradigm: contextual aversive conditioning

All participants underwent a differential contextual aversive conditioning protocol twice, 1 week apart, but at the same time of day (between 1:00 pm and 4:00 pm) following the SECPT or the control procedure. We used two parallel versions of a contextual conditioning paradigm balanced according to the preceding exposure. An aversive sound served as the US: an aversive scream from the International Affective Digital Sounds (IADS, Bradley and Lang, 1999), Sound No. 276, or an aversive scraping over slate (Neumann et al., 2008). Each US was presented for 2 s. Two complex environments (3D pictures of similar-looking rooms with a slightly different furniture arrangement) served as contextual conditioned stimuli (CON1 and CON2). Assignment of CON1 and CON2 was balanced according to the preceding exposure. Each CON was presented for 10 s. The intertrial interval varied between 8 and 10 s.

**Figure 1:**
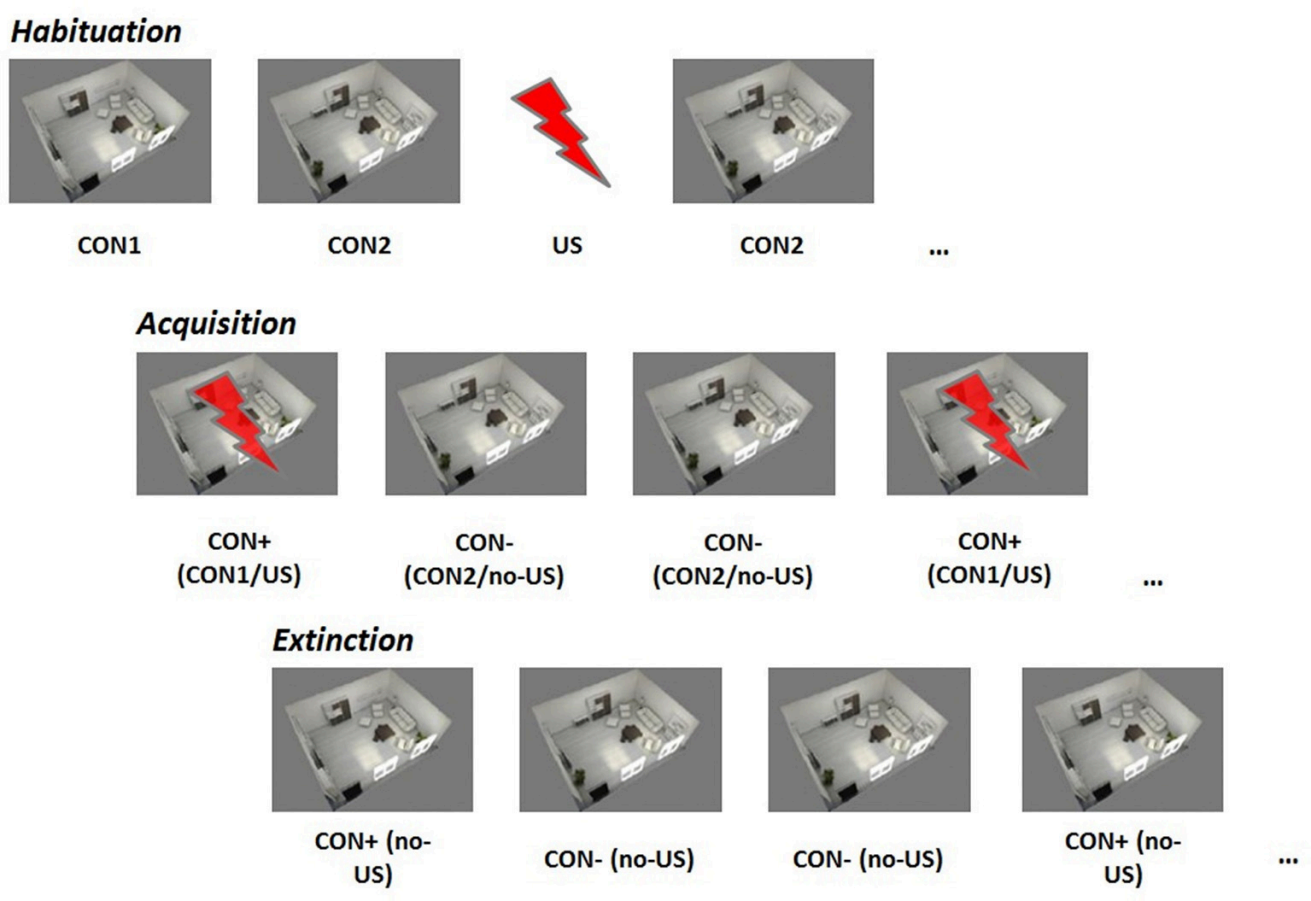
**Contextual fear conditioning paradigm**. CON1/+, conditioned stimulus paired with the unconditioned stimulus; CON2/−, conditioned stimulus never paired with the unconditioned stimulus, US, unconditioned stimulus.

Figure 1 gives a schematic overview of the three paradigm phases.

Habituation: During the habituation phase, each stimulus (CON1 / CON2 / US) was presented separately six times in a randomized order.

Acquisition: During the acquisition phase, 12 CON1-US pairings (CON+) and 12 CON2 alone (CON−) were delivered in a randomized order. For CON+, CON1 was paired with the US in 100%.

Extinction: During the extinction phase, 8 CON+ and 8 CON− were presented in a randomized order. No US was delivered during this phase.

Ratings of valence, arousal, and contingency of all stimuli were assessed after the habituation, the acquisition and the extinction phase. For valence and arousal we used the Self-Assessment Manikin (SAM; Bradley and Lang, 1994), which was subsequently transformed to a numerical scale ranging from 1 point to 9 points. Contingency was assessed with the question “how likely was this room accompanied by an aversive sound” on a 9-point scale ranging from “extremely unlikely” to “extremely likely” to indicate the suspected degree of CS-US contingency. We excluded 5 individuals (*n* = 3 TGA and *n* = 2 controls) from further analysis who had already rated the contexts as aversive during habituation (value above 5 on valence and arousal) and thus no longer fulfilled the CS criteria of being neutral before the learning phase.

### fMRI: examination parameters, data processing, and data analysis

The MRI session was performed on a 3 T MAGNETOM Skyra whole body MR scanner (Siemens Medical Solutions, Erlangen, Germany) using a 20-channel head/neck coil.

#### Functional imaging

Blood oxygenation level-dependent (BOLD) whole-brain functional images were acquired using a T2^*^-weighted gradient-echo echo planar imaging (EPI) sequence (TR = 2210 ms, TE = 23 ms, FoV = 220 × 220 mm^2^, matrix size = 96 × 96, voxel size = 2.3 × 2.3 × 2.3 mm^3^, flip angle = 90°, bandwidth = 1270 Hz/px, parallel acquisition technique GRAPPA acceleration factor 2). For each image volume 36 axial slices (slice thickness = 2.3 mm, gap = 0.7 mm) were recorded in descending order, positioned along the anterior and posterior commissure (AC-PC orientation).

#### Data processing and analysis

fMRI data analysis was performed using Statistical Parametric Mapping software (SPM8, Wellcome Department of Imaging Neuroscience, London, UK) implemented on MATLAB R2010a (The MathWorks Inc., Natick, MA, USA). Before pre-processing, we discarded the first three volumes of each scanning session to allow for T_1_ equilibration. We conducted standard spatial and temporal pre-processing: realignment to the first volume using a rigid body transformation to correct for head movements, and slice time correction to reference slice 18. None of the participants had to be excluded from the analysis due to excessive movement (motion cut-off criteria: greater than 2 mm in translation, 2 degrees in rotation). Normalization was done using the Montreal Neurological Institute International Consortium for Brain Mapping (MNI) space, smoothing with a 7 × 7 × 9 mm^3^ full-width at half-maximum Gaussian kernel, and high-pass filtering with a cut-off 1/128 Hz to remove low-frequency noise and corrected for serial autocorrelations using first-order autoregressive functions AR. We performed a fixed effects analysis using a general linear model including the following experimental regressors: CON1, CON2, and US for habituation, CON+ and CON− for acquisition and CON+ and CON− for extinction. These regressors were convolved with a canonical hemodynamic response function (first order expansion) to create the design matrix and we added the six movement parameters resulting from the rigid body transformation as confound variables. At group level, we used a random effects analysis to analyse the contrast of interest (CON+ > CON−). We applied a regions of interest (ROIs) approach using the MNI template Automated Anatomical Labeling (Tzourio-Mazoyer et al., 2002), implemented in the Wake Forest Pick Atlas (Maldjian et al., 2003). We analyzed the following ROIs: hippocampus, amygdala, insula, ACC, PFC, striatum (Lang et al., 2009; Pohlack et al., 2012), and the significance level was set to *p* < 0.05, corrected for multiple comparisons using a family-wise error (FWE) rate. Moreover, we used anxiety, depression and cortisol levels as covariates of no interest in the analyses to control for possible effects of these variables on acquisition and extinction, because it is known that they can directly affect conditioning (Sehlmeyer et al., 2011; Merz et al., 2013b).

### Statistical analysis

For the cortisol responses during the SECPT and control procedure we used a repeated measures analysis of variance (rmANOVA) with “time point” (three levels: before, 3 min, 15 min) as the within-subject and “group” (two levels: TGA, controls) as the between-subject factor. Moreover, we also analyzed these data from the SECPT and the control procedure within each group before vs. after the SECPT or control procedure using an rmANOVA with “time point” (three levels: before, 3 min, 15 min) as the within-subject factor for each group. For the subjective ratings (valence, arousal, contingency) during contextual conditioning, we used an rmANOVA for each phase (habituation, acquisition, extinction) using “stimulus” (two levels: CON+, CON−) as the within-subject and “group” (two levels: TGA, controls) as the between-subject factor, for both the SECPT and the control procedure. Moreover, we analyzed our conditioning data also within the groups using paired sample *t*-tests looking for differences in response to the CON+ vs. CON− to determine possible learning alterations in each group. For the subjective ratings during contextual conditioning, we used anxiety, depression and cortisol levels as covariates in the analyses, in accordance with the fMRI analysis. For all ANOVAs, follow-up *t*-tests were conducted following Bonferroni-correction. Whenever the assumption of homogeneity of variances was violated, we applied the Greenhouse–Geisser adjustment and corrected degrees of freedom are reported. The significance level for all statistical tests was set to *p* < 0.05. For all analyses, we used the Predictive Analytics Software release 18.0.1 (PASW, SPSS Inc., Chicago, IL).

## Results

### Cortisol response following control procedure and SECPT

#### Control procedure

Neither the TGA nor the control group showed significant differences in cortisol before vs. after the control procedure. Although before and 3 min after the procedure, TGA individuals showed increased cortisol levels compared to the control individuals, 15 min after the procedure and thus immediately before the contextual aversive conditioning, the cortisol levels of the two groups were not significantly different (see Table 2).

**Table 2:** **Cortisol response during the SECPT and the control procedure in TGA individuals vs. healthy controls**.

	**TGA**	**Control group**	***p*-value**
**CONTROL PROCEDURE**			
Before	10.53 (0.97)	8.14 (0.61)	0.022
3 min after	10.77 (1.08)	8.23 (0.59)	0.024
15 min after	8.89 (0.79)	7.75 (0.64)	n.s.
**SECPT**			
Before	11.28 (1.44)	7.12 (0.75)	0.008
3 min after	11.81 (1.34)	7.92 (0.86)	0.010
15 min after	13.72 (2.08)	12.25 (1.41)	n.s.

*Data are presented in nmol/l; mean (standard error of the mean); n.s., non-significant; TGA, transient global amnesia; SECPT, Socially Evaluated Cold Pressor Test*.

#### SECPT

Both the TGA and the control group showed a significant increase in cortisol values in response to the stress exposure, i.e., before the SECPT vs. 15 min following the SECPT. Moreover, before and 3 min after the procedure TGA individuals showed increased cortisol levels compared to the control individuals, however, at 15 min after the procedure and thus directly before the contextual aversive conditioning fMRI, the cortisol levels of TGA patients and controls were not significantly different (see Table 2).

#### SECPT vs. control procedure

In both groups, cortisol values immediately before the contextual aversive conditioning fMRI were significantly higher after the SECPT than after the control procedure (TGA: *p* = 0.014; controls: *p* = 0.004).

Further details on cortisol and sympathetic response during the SECPT and control procedure have been reported recently (Griebe et al., 2015).

In summary, participants with a previous TGA showed significantly higher cortisol levels compared to the control group before and after both the control procedure and the experimental stressor. However, after each experiment and immediately before fMRI, the cortisol levels of TGA patients and controls were not significantly different.

### Subjective ratings of conditioned stimuli during fMRI

#### Control group

**Figure 2:**
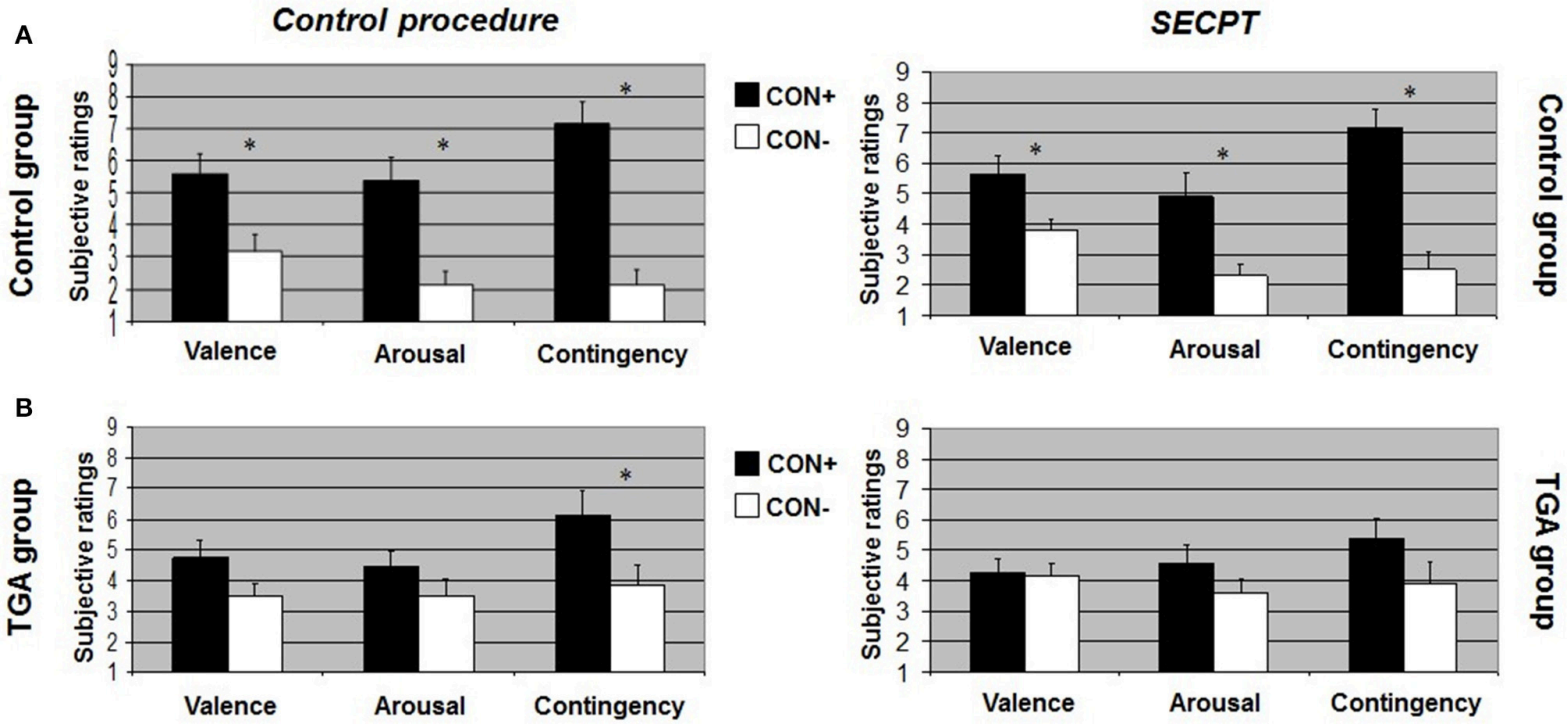
**Subjective ratings of valence, arousal and contingency during the acquisition phase of the contextual fear conditioning paradigm. (A)** In the control group, **(B)** in the TGA group, following the control procedure and the SECPT. ^*^p < 0.05. CON+, conditioned stimulus paired with the unconditioned stimulus; CON−, conditioned stimulus never paired with the unconditioned stimulus; SECPT, Socially Evaluated Cold Pressor Test; TGA, transient global amnesia.

**Figure 3:**
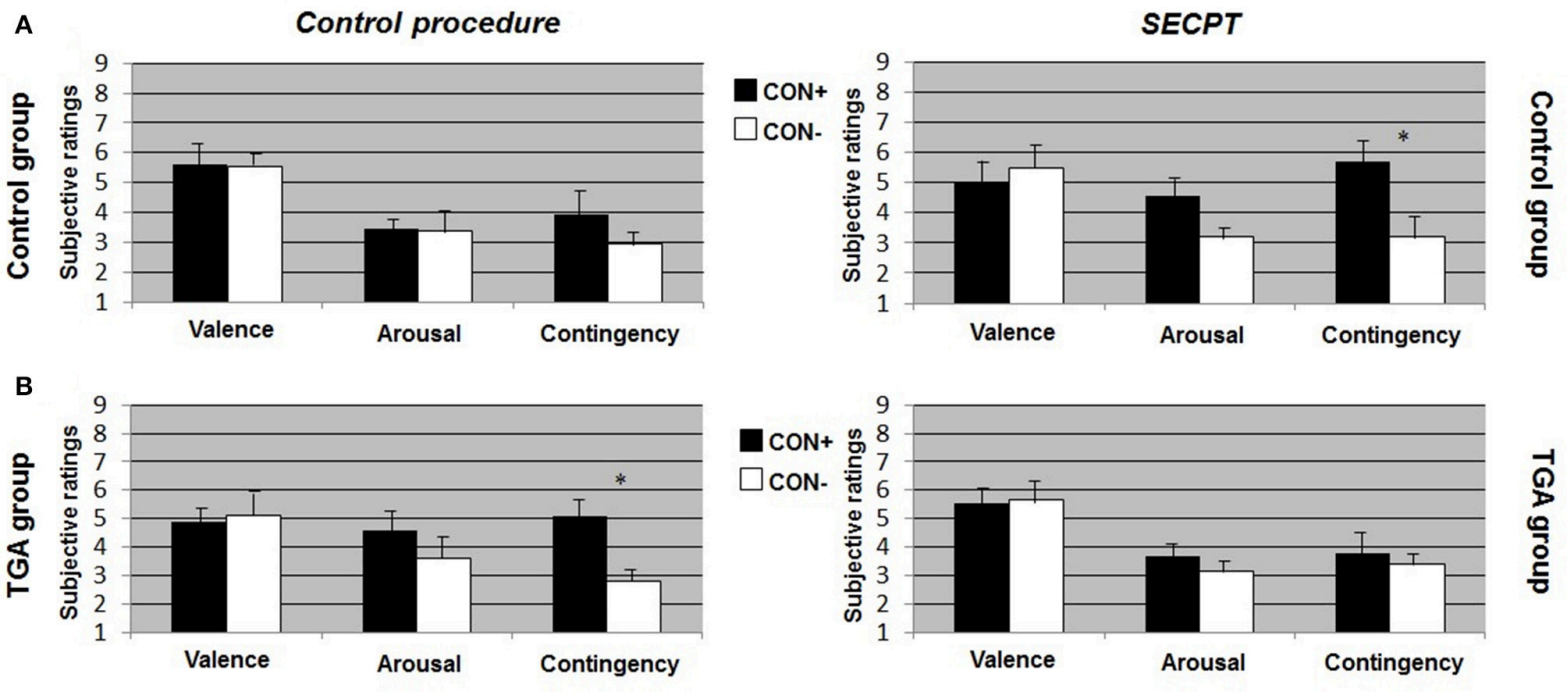
**Subjective ratings of valence, arousal and contingency during the ***extinction*** phase of the contextual fear conditioning paradigm. (A)** In the control group, **(B)** in the TGA group, following the control procedure and the SECPT. ^*^p < 0.05. CON+, conditioned stimulus paired with the unconditioned stimulus; CON−, conditioned stimulus never paired with the unconditioned stimulus; SECPT, Socially Evaluated Cold Pressor Test; TGA, transient global amnesia.

After the *control procedure*, we found no significant differences between CON1 and CON2 following habituation, neither for valence, arousal or contingency ratings. Following acquisition, control individuals showed significantly increased valence [*t*_(17)_ = 2.551, *p* = 0.021], arousal [*t*_(17)_ = 4.164, *p* = 0.001] and contingency [*t*_(17)_ = 5.846, *p* < 0.001] ratings to CON+ vs. CON− (see Figure 2A, left side). Following extinction, significant differences between CON+ and CON− were no longer detectable in any of the ratings (see Figure 3A, left side). After the *SECPT*, we found no significant differences between CON1 and CON2 for any of the ratings following habituation. Following acquisition, control individuals showed significantly increased valence [*t*_(17)_ = 2.188, *p* = 0.042], arousal [*t*_(17)_ = 2.807, *p* = 0.012] and contingency [*t*_(17)_ = 4.426, *p* < 0.001] ratings to CON+ vs. CON− (see Figure 2A, right side). Following extinction, significant differences between CON+ and CON− for valence and arousal ratings were no longer detectable, but control individuals still showed significantly increased contingency ratings to CON+ vs. CON− [*t*_(17)_ = 2.825, *p* = 0.012] (see Figure 3A, right side). Last, for ratings of valence, arousal and contingency following habituation, acquisition and extinction, we found no significant differences between the *SECPT* and the *control procedure*.

#### TGA group

After the *control procedure*, we found no significant differences between CON1 and CON2 in the ratings following habituation. Following acquisition, TGA individuals did not show significant differences between CON+ and CON− for valence and arousal ratings, but significantly increased contingency ratings [*t*_(17)_ = 2.231, *p* = 0.043] in response to CON+ vs. CON− (see Figure 2B, left side). Following extinction, TGA individuals still showed significantly increased contingency ratings [*t*_(17)_ = 2.112, *p* = 0.05] in response to CON+ vs. CON−, yet no significant differences for valence and arousal ratings (see Figure 3B, left side). After the *SECPT*, we found no significant differences between CON1/+ and CON2/− in any of the ratings following habituation, acquisition (see Figure 2B, right side) or extinction (see Figure 3B, right side). Moreover, we observed no significant differences between the *SECPT* and *control procedure* in the ratings after each phase.

#### TGA vs. control group

After the *control procedure*, we found significant differences between the groups for arousal [interaction effect of “stimulus” × “group”: *F*_(1, 35)_ = 4.940, *p* = 0.033] and contingency ratings [interaction effect of “stimulus” × “group”: *F*_(1, 35)_ = 4.449, *p* = 0.043] to CON+ vs. CON− following acquisition. The difference between CON+ vs. CON− was significantly larger in the control group compared to the TGA group for both arousal (*p* = 0.033) and contingency (*p* = 0.043). After the *SECPT*, we found significant differences between the groups only for contingency ratings [interaction effect of “stimulus” × “group”: *F*_(1, 35)_ = 4.126, *p* = 0.05] following acquisition, with a greater difference between CON+ vs. CON− in the control compared to TGA individuals (*p* = 0.05).

In summary, controls showed successful acquisition of conditioned responses after the control procedure, and also successful extinction of the conditioned responses. Following stress, acquisition was still successful, however extinction was impaired. In contrast, TGA patients showed an impairment of conditioned responses already during the acquisition phase of the control procedure for valence and arousal ratings but increased contingency ratings. This rating remained unchanged after the extinction phase of the control procedure. After stress, no significant learning was found.

### Brain activation during contextual aversive conditioning fMRI

**Figure 4:**
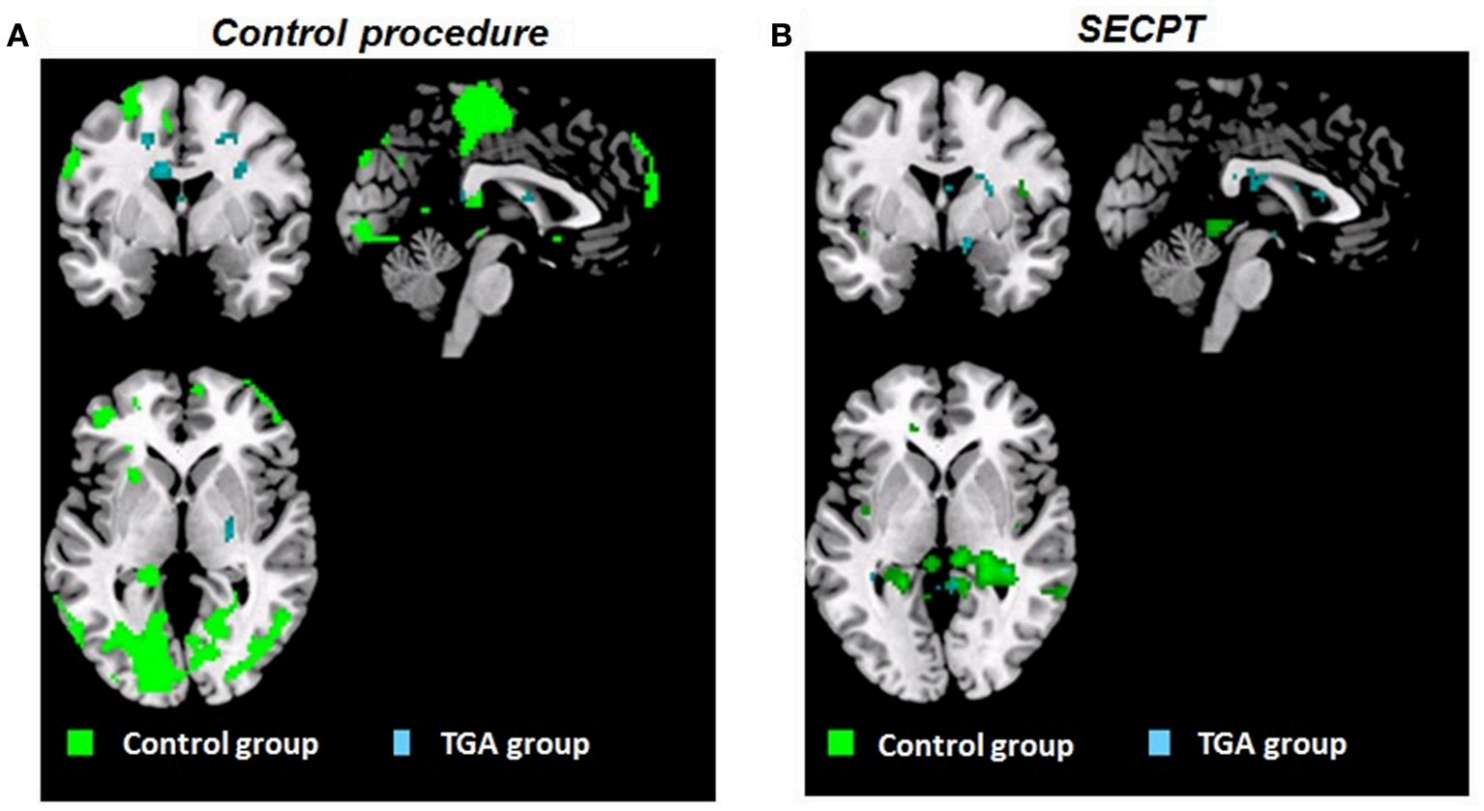
**Whole brain activation patterns (***p***_**FWE**_ < 0.05)**. Contrast CON+ > CON− during the *acquisition* phase of the contextual fear conditioning paradigm **(A)** following the control procedure, **(B)** following the SECPT. Activation in control group indicated in green, TGA group in blue. CON+, conditioned stimulus paired with the unconditioned stimulus; CON−, conditioned stimulus never paired with the unconditioned stimulus; SECPT, Socially Evaluated Cold Pressor Test; TGA, transient global amnesia.

Figure 4 shows an overview of brain activation patterns during the acquisition phase in the TGA and control group following the SECPT and the control procedure.

#### Control group

Following the *control procedure*, we did not observe any significant differences in the brain response to CON1 vs. CON2 during habituation and to CON+ vs. CON− during extinction, neither on the whole brain level nor for our pre-defined ROIs. During acquisition, we observed significantly increased whole brain responses in an occipital lobe cluster (medial and inferior surfaces—left lingual gyrus; lateral surfaces—left superior occipital gyrus) and a frontal lobe cluster (medial surface—right paracentral lobule). In our pre-defined ROI analysis, we found significantly increased responses in the left hippocampus (*x* = −20, *y* = −38, *z* = 4; *T* = 15.13; *p*_FWE_ < 0.006), but not in the amygdala, insula, ACC, PFC and striatum. Following the *SECPT*, we observed no significant differences in the brain response to CON1 vs. CON2 during habituation and to CON+ vs. CON− during extinction, neither voxel-wise at whole brain level nor for our pre-defined ROIs. During acquisition, we no longer observed a significant response in the hippocampus to CON+ vs. CON−, nor in any other ROI or at whole brain level. Comparing brain responses in the *SECPT* vs. *the control procedure*, we observed no significant differences in any of the conditioning phases.

#### TGA group

**Figure 5:**
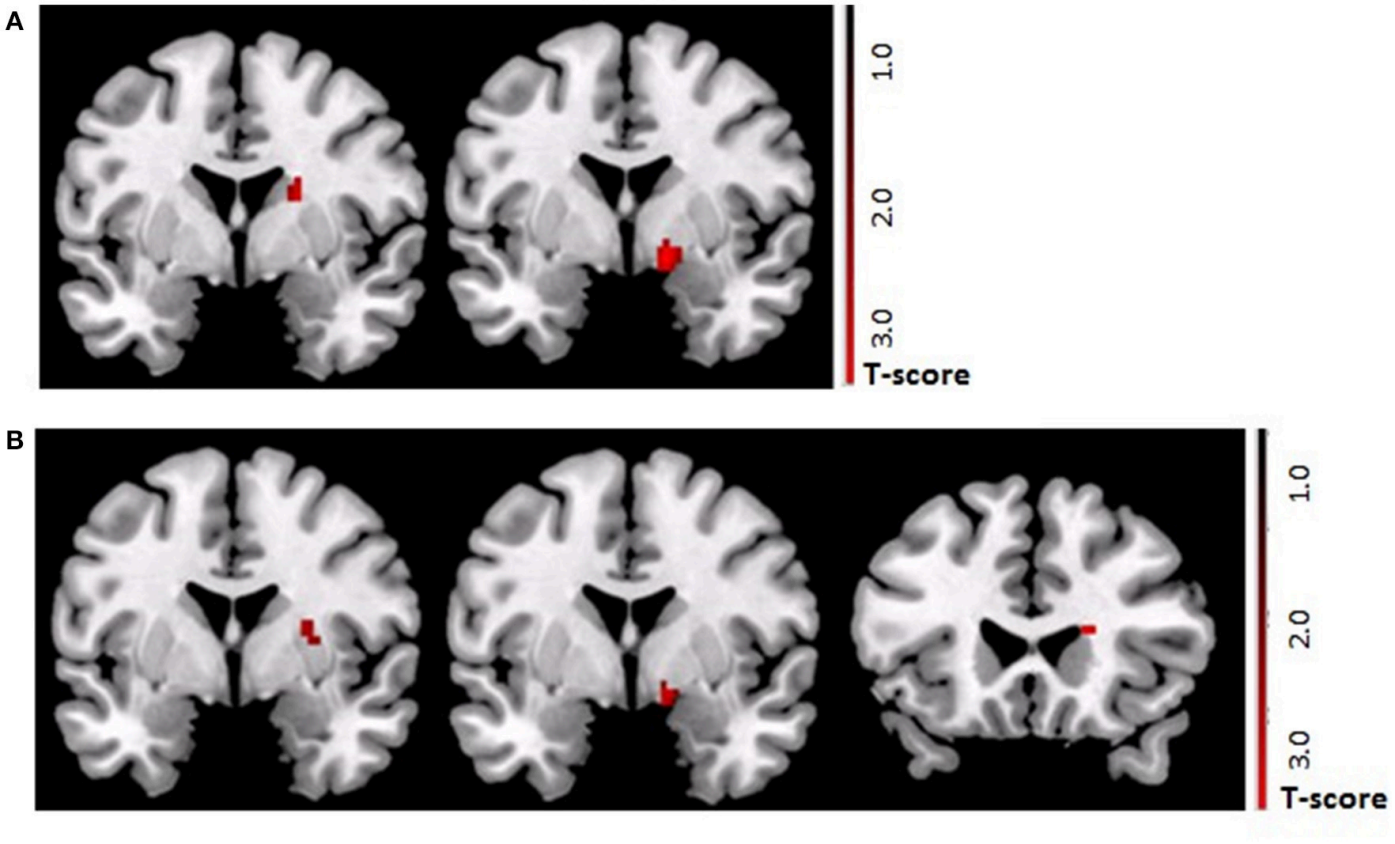
**(A)** ROI based analysis of brain activation (*p*_FWE_ < 0.05) in the TGA group. Contrast SECPT > control procedure during *acquisition* phase (CON+ > CON−) of the contextual fear conditioning paradigm showing a stronger activation of the right ventral striatum (*x* = 19, *y* = 3, *z* = −5; *T* = 3.83) after the SECPT. **(B)** ROI based analysis of brain activation (*p*_FWE_ < 0.05) following the SECPT. Contrast TGA > control group during the *acquisition* phase (CON+ > CON−) of the contextual fear conditioning paradigm showing a stronger activation of the right ventral striatum (*x* = 19, *y* = 17, *z* = −11; *T* = 3.53) in the TGA group. ROI, region of interest; CON+, conditioned stimulus paired with the unconditioned stimulus; CON−, conditioned stimulus never paired with the unconditioned stimulus; SECPT, Socially Evaluated Cold Pressor Test; TGA, transient global amnesia.

Following the *control procedure*, we observed no significant brain responses neither to CON1 vs. CON2 during habituation nor to CON+ vs. CON− during acquisition and extinction. Following the *SECPT*, we did not observe any significant brain responses to CON1 vs. CON2 during habituation and to CON+ vs. CON− during extinction. However, during acquisition we observed an increased response in the right striatum to CON+ vs. CON− (*x* = 21, *y* = 8, *z* = −2; *T* = 4.15; *p*_FWE/trend_ = 0.06), but no significant differences in any other ROI or voxel-wise at whole brain level. Comparing brain responses in the *SECPT* vs. *the control procedure*, we observed a significantly increased response in the right ventral striatum (*x* = 19, *y* = 3, *z* = −5; *T* = 3.83; *p*_FWE_ = 0.031; see Figure 5A) to CON+ vs. CON− during acquisition, but no significant effects for habituation and extinction.

#### Control vs. TGA group

Following the *SECPT*, we observed no significant effects for habituation and extinction, but a significantly increased response in the striatum to CON+ vs. CON− during acquisition in the TGA compared to the control group (*x* = 19, *y* = 17, *z* = −11; *T* = 3.53; *p*_FWE_ = 0.03; see Figure 5B).

In summary, controls showed an overall increase in brain activation pattern and an increased response in the hippocampus during acquisition. This was not observed after the experimental stressor. In TGA patients, we did not observe a change in the overall activation pattern after either condition; however, an increased response was found in the right ventral striatum in the acquisition phase following stress.

## Discussion

The aim of the present study was to investigate the effect of experimentally induced stress on aversive conditioning related brain activation in patients with a recent history of TGA compared to a control group. First, we found that both the control and the TGA patients exhibited significantly increased cortisol values and stress ratings following the SECPT when compared to the values of both groups after the control procedure. Second, we observed significant alterations in fear conditioning in TGA compared to controls.

After the SECPT, learning on a neural level was impaired in the control group, indicated by a reduction in hippocampal as well as frontal and occipital lobe cluster brain activation. This fits with previous studies that have observed stress- and glucocorticoid-dependent decreases in hippocampal responsivity during declarative memory and learning (Schwabe and Wolf, 2012). On a subjective level, our control participants were still able to differentiate CON+ and CON−, as demonstrated by significantly increased valence, arousal and contingency ratings to CON+ vs. CON−. Extinction learning featured significantly reduced contingency awareness. So far, only a few studies have addressed the role of stress on conditioning (Merz et al., 2013b). One of these studies found an increased CON+/ CON− differentiation in the hippocampus following stress in healthy participants, additionally mediated by significant effects of sex (Merz et al., 2013b). These studies used cue, not contextual conditioning procedures, which makes our findings not directly comparable to this previous literature. Moreover, the population in the present study consisted of elderly subjects, for which, to the best of our knowledge, only one human study on conditioning exists so far (Cuppini et al., 2006). In this study, the authors found reduced differential learning and contingency awareness in middle-aged and older compared to young individuals. However, the role of stress in this context was not investigated. Future research on the effects of age on contextual conditioning following stress exposure therefore needed to further classify our present findings in a respective fashion.

The TGA group showed no successful contextual acquisition after the control procedure, indicated by non-significant differences between CON+ and CON− for valence and arousal ratings and significantly extenuated differentiation between CON+ and CON− for arousal and contingency ratings compared to the control group as well as by no significant differences in hippocampal activation to CON+ vs. CON−. We also observed a reduced extinction learning in the TGA group, evidenced by contingency ratings that were still increased in response to CON+ vs. CON− following the extinction phase. Therefore, while the control group showed alterations in context conditioning only following the SECPT, the TGA group might be characterized by analogous impairments already after the non-stress control procedure and thus under normal circumstances.

Also, after the SECPT, the TGA compared to the control group revealed a progressive impairment of fear learning, and, compared to the control procedure, a normalization of extinction learning, i.e., successful extinction, with no longer significantly different contingency ratings between CON+ vs. CON−. This might be interpreted as a consequence of the inferior acquisition learning: since they no longer learned to differentiate CON+ from CON−, there was no need to extinct this information. On a neural level, we identified a significantly increased activation in the striatum in the TGA compared to the control group after the SECPT and also within the TGA group as a response to stress exposure vs. the non-stress control procedure. The striatum is a brain region known to be involved in anticipation and prediction, and thus evaluation of processes during learning (Li et al., 2011; Pohlack et al., 2012). This might indicate a shift in the brain in that other brain regions cover the function of the hippocampus when its response is reduced. Therefore, TGA individuals might be more engaged in anticipating negative events and thus may invest more effort to evaluate their environment following stress. As we investigated individuals who had experienced a TGA in the last 2 years, these processes might be interpreted as coping strategies developed because of their previous TGA experience. While the hippocampus may serve as target brain region without any relation to stress in TGA, the striatum may serve as brain area that is directly related to the experience and in consequence to the processing of stress in TGA. A specific role of the striatum in conditioning following acute stress exposure was recently demonstrated for Pavlovian cue conditioning with monetary reinforcers (Lewis et al., 2014) and in memory and instrumental behavior (Schwabe and Wolf, 2012). Future studies are needed to shed further light into these mechanisms in TGA—specifically the investigation of individuals in the acute stage of TGA might provide further important information.

Our findings raise the question, which possible factors might be associated with the observed general contextual learning impairments in TGA. Many studies have suggested a crucial role of the hippocampus in tasks involving learning and remembering contexts (Holland and Bouton, 1999). Experimental studies have reported that hippocampal lesions cause deficits in freezing behavior during exposure to a shock-paired context if the lesions are produced after conditioning (Phillips and LeDoux, 1992). However, these animals use context information less efficiently due to a dysfunction in context encoding (Wiltgen et al., 2006). Thus, although we did not observe significant functional alterations in the hippocampus during context conditioning in TGA compared to controls, the short-lasting hippocampal lesions during the acute phase of the TGA episode might have (co-) determined the impairments in context conditioning in TGA following both stress exposure and the control procedure. Interestingly, hippocampal lesions do not necessarily interrupt context fear conditioning when they are present before conditioning (Maren et al., 2013). This might underline our suggestions. Another factor might be the situation in the hospital, i.e., the whole research context might have been experienced as an extremely negative environment, possibly due to the previous experiences during the acute phase of the TGA episode. This situation might have served as strong aversive context and therefore as so called second-order US, a process known from second order conditioning or cue reactivity procedures. Here, a personally relevant emotional stimulus or event, for example a traumatic event in patients with posttraumatic stress disorder (Wessa and Flor, 2007; Diener et al., 2016) or drug-related stimuli in patients with drug addiction (Carter and Tiffany, 1999), acquire the qualities of an US based on learning principles, and may generalize to neutral stimuli. Consequences could be a reduction in extinction learning, as for example demonstrated in posttraumatic stress disorder patients (Diener et al., 2016), or in stronger reactions to disorder- and personally relevant contextual stimuli, as demonstrated in abstinent alcoholics, who showed significantly reduced startle reflex magnitudes in response to social and pub contexts compared to neutral contexts, although the drug alcohol was present in all these situations (Nees et al., 2012). Therefore, not only a single stimulus may drive behavior, but also the context may represent an important modulating factor that can determine our responses and subsequent behavior (Flor and Nees, 2014). Our finding of an anticipatory cortisol response (Griebe et al., 2015) in the TGA group might be interpreted as an implication of such an effect.

Moreover, along the assumptions on the existence of multiple memory systems, the hippocampus and the striatum are supposed to represent key divergent brain regions that serve different levels of the memory process. Whereas, the hippocampus is assumed to be mainly recruited during cognitive engagement and purposeful memory activation, there is a shift to dorsal striatal areas when memory functions turn into habits, i.e., more automatic processes that require little attention and effort (Packard and Goodman, 2012). Stress and anxiety have been shown to enhance dorsal striatal-dependent habit memory, at the expense of hippocampal-dependent cognitive memory possibly via modulating activity of the basolateral amygdala. This mechanism is believed to have implications for the development of certain psychiatric disorders, such as posttraumatic stress disorder (Packard, 2009). Our findings imply that such a shift from a hippocampus-dependent to a striatum-dependent strategy was stronger in the TGA patients—as a possible consequence of hippocampal impairment.

The present findings should be seen in light of some limitations. We did not assess a peripheral measure of conditioning such as skin conductance responses, which may differ from the verbal ratings (Birbaumer et al., 2005) and would have allowed us to use another measure captured over the course of the conditioning procedure for the interpretation of the data. Moreover, our findings could have been affected by sex, which was reported in previous conditioning studies (Milad et al., 2006), for example based on potential influences of sex hormones (Merz et al., 2012). However, our sample was matched for age and sex. Last, it needs to be mentioned that our finding of hippocampal impairment in TGA was inferred from *t*-tests comparing hippocampal response to CON+ vs. CON− separately in each group and not from a significant group effect. One could argue that this significant finding in one group but not the other group is insufficient evidence for suggesting different task-related activity between groups, as mean activity in one group may be just below the critical threshold and in the other group just above. However, while we found reduced response in the hippocampus in TGA individuals (although being not significant), hippocampal response was increased in control individuals. This speaks against a possible near-threshold effect. The non-significant group effect for hippocampal response might be due to our small sample size.

In sum, we found evidence for impairments in implicit learning processes, both in relation to stress, but also independent of stress experience, in individuals who had suffered a TGA episode. This finding might reflect a general pathological characteristic of TGA. The implicit learning impairments can either precede a TGA episode or be a consequence of the disorder, which should be further addressed in future studies by assessing individuals within the acute phase of TGA.

## Author contributions

FN: Study conception and design, data collection and analysis, interpretation, drafting and revising the manuscript. MG: Study conception and design, data collection and analysis, interpretation, drafting and revising the manuscript. AE: Data collection and analysis, revision of the manuscript. MR: Data collection and analysis, revision of the manuscript. BG: Data collection and analysis, revision of the manuscript. OW: Data analysis, interpretation, revision of the manuscript. LS: Data analysis, interpretation, revision of the manuscript. AG: Study conception and design, data analysis, revision of the manuscript. KS: Study conception and design, data collection and analysis, interpretation, drafting and revising the manuscript. All authors have approved of the final manuscript version to be published and have agreed to be accountable for all aspects of the work in ensuring that questions related to the accuracy or integrity of any part of the work are appropriately investigated and resolved.

## Funding

Funding for this study was provided by project C07/Z03 of the Deutsche Forschungsgemeinschaft (DFG) Collaborative Research center 636 “Learning, memory and brain plasticity: Implications for psychopathology.”

### Conflict of interest statement

FN, LS, and KS have received funding for this study from the Deutsche Forschungsgemeinschaft (DFG); project C07/Z03 of the Collaborative Research center 636 “Learning, memory and brain plasticity: Implications for psychopathology.” All other authors declare that the research was conducted in the absence of any commercial or financial relationships that could be construed as a potential conflict of interest.

## Abbreviations

ADS: General Depression Scale (Allgemeine Depressionsskala)
HPA axis: Hypothalamic pituitary adrenal axis
SECPT: Socially evaluated cold pressor test
STAI: State-Trait Anxiety Inventory
TGA: Transient global amnesia
TICS: Trier Inventory for Chronic Stress.
